# Preoperative Zoledronate Use and Risk of Postoperative Hypocalcemia After Parathyroidectomy for Primary Hyperparathyroidism: A Comparative Retrospective Cohort Study

**DOI:** 10.3390/jcm15114080

**Published:** 2026-05-25

**Authors:** Ümit Çavdar, Mustafa Duman, Özlem Eren, Mehmet Sercan Ertürk, Serkan Karaıslı, Emine Özlem Gür, Selda Gücek Haciyanli, Barış Önder Pamuk, Mehmet Haciyanli

**Affiliations:** 1 Division of Endocrinology and Metabolism, Atatürk Training and Research Hospital, Katip Çelebi University, 35360 Izmir, Türkiye; duman.mustafa35@hotmail.com (M.D.); ozlemeren138@gmail.com (Ö.E.); msercanerturk@gmail.com (M.S.E.); bopamuk@gmail.com (B.Ö.P.); 2 Division of General Surgery, Atatürk Training and Research Hospital, Katip Çelebi University, 35360 Izmir, Türkiye; skaraisli@hotmail.com (S.K.); eozlemgur@yahoo.com (E.Ö.G.); msgucek@hotmail.com (S.G.H.); mhaciyanli@hotmail.com (M.H.)

**Keywords:** hyperparathyroidism, primary, zoledronic acid, bisphosphonates, hypocalcemia, hungry bone syndrome, parathyroidectomy

## Abstract

**Background:** Given the limited and conflicting evidence regarding both the role of bisphosphonate (BP) use and the risk factors for postoperative hypocalcemia, as well as ongoing clinical hesitation toward preoperative BP use due to concerns about the occurrence of hypocalcemia, we aimed to investigate their association in patients with primary hyperparathyroidism (PHPT). **Materials and Methods:** A total of 73 patients undergoing parathyroidectomy were included, with 22 in the BP-positive (zoledronate) group and 51 in the BP-negative group. In BP-treated patients, biochemical values obtained after zoledronate infusion but before surgery were used as the preoperative baseline for perioperative comparisons, whereas pre-infusion calcium values were used only to characterize the clinical indication for treatment. Factors associated with postoperative hypocalcemia were evaluated using hypocalcemia as a dichotomous outcome. **Results:** Postoperative hypocalcemia was more frequent in BP-treated patients (27.3% vs. 3.9%, *p* = 0.008). However, in multivariable analysis including alkaline phosphatase (ALP), the association between BP use and hypocalcemia was attenuated and no longer statistically significant (OR 4.81, 95% CI 0.74–31.30; *p* = 0.100). ALP was independently associated with hypocalcemia (OR 1.016, 95% CI 1.001–1.031; *p* = 0.040). Additionally, hypocalcemia was associated with a shorter infusion-to-surgery interval (median 10 vs. 72 days, *p* = 0.0045) among BP-treated patients. **Conclusions:** Although hypocalcemia was more frequent among patients receiving preoperative zoledronate, this association was attenuated after adjustment for alkaline phosphatase and may reflect underlying disease severity. Alkaline phosphatase was independently associated with postoperative hypocalcemia, supporting the role of increased bone turnover. Given the limited number of events, an independent effect of zoledronate cannot be excluded, and treatment timing may warrant further investigation.

## 1. Introduction

Primary hyperparathyroidism (PHPT) is a common endocrine disorder with an overall prevalence of 1.08% [1]. Parathyroidectomy is indicated in patients with primary hyperparathyroidism who have significant hypercalcemia, impaired renal function, renal calculi or nephrocalcinosis, hypercalciuria, osteoporosis, or fragility fractures, as well as in those younger than 50 years [2]. However, medical management may be considered in patients with mild asymptomatic disease, contraindications to surgery, or failed prior interventions [3]. In PHPT, bisphosphonate (BP) therapy may be the treatment of choice for patients presenting with hypercalcemia and/or concomitant osteoporosis. Meta-analyses comparing medical management with surgery in mild PHPT have demonstrated stable calcium but rising parathyroid hormone (PTH) levels in the medically treated groups [4]. BPs, including alendronate and zoledronic acid, are antiresorptive agents shown to improve bone mineral density (BMD) in patients with primary hyperparathyroidism, accompanied by an initial reduction in serum calcium levels lasting up to six months. However, alendronate use has been associated with a subsequent increase in parathyroid hormone (PTH) levels above baseline [5]. Nonetheless, parathyroidectomy remains the mainstay of treatment, offering superior improvements in BMD, reduction in renal stone formation, and preservation of renal function compared to medical therapy [3,4].

Postoperative hypocalcemia is a well-recognized complication of parathyroid surgery, occurring in up to 38.4% of patients on the first postoperative day [6]. This early biochemical hypocalcemia encompasses a spectrum ranging from mild and transient reductions in serum calcium to clinically significant forms, including hungry bone syndrome (HBS), early severe hypocalcemia, and hypocalcemia related to transient or permanent hypoparathyroidism.

In cases with HBS, symptoms of neuromuscular irritability, such as paresthesia, carpopedal spasm, laryngospasm, seizures, and tetany typically manifest within 1–3 days after surgery with low serum calcium levels (<8.5 mg/dL) lasting more than 3 days postoperatively [6,7,8]. It is followed by a prolonged period of milder hypocalcemia accompanying hypophosphatemia and hypomagnesemia, which may persist for months [9,10]. Risk factors include older age, large adenoma size, high preoperative PTH and alkaline phosphatase (ALP) levels, skeletal involvement, osteoporosis, and parathyroid hyperplasia [6,11].

Early severe hypocalcemia, by contrast, refers to a rapid decline in serum calcium (<7.6 mg/dL) within the first 18 h postoperatively, often requiring urgent intravenous replacement [8,9].

Hypoparathyroidism constitutes another important cause of postoperative hypocalcemia and is characterized by inappropriately low or undetectable PTH levels in the setting of hypocalcemia. This typically reflects inadvertent parathyroid removal or vascular compromise during surgery [7].

Despite concerns regarding hypocalcemia, preoperative bisphosphonate therapy may facilitate safer anesthetic and perioperative management by controlling severe hypercalcemia. In addition, biochemical stabilization may provide valuable time for comprehensive preoperative localization studies and patient optimization, given that severe hypercalcemia can substantially compromise anesthetic safety and perioperative stability [12].

However, the role of preoperative bisphosphonate use in primary hyperparathyroidism remains controversial. Bisphosphonates have been shown to stabilize skeletal turnover, improve bone mineral density, and potentially mitigate postoperative hypocalcemia [13,14] with some studies reporting reduced rates of hungry bone syndrome and shorter hospital stays [14,15], whereas others have described cases of hungry bone syndrome despite preoperative therapy [16,17].

While bisphosphonates have been used to prevent hungry bone syndrome in secondary hyperparathyroidism [8], evidence supporting their use in primary hyperparathyroidism remains limited, with most available data derived from small, non-comparative cohorts. Consequently, the clinical impact of this approach remains insufficiently defined.

In this context, the present study aimed to evaluate whether preoperative bisphosphonate use is associated with the risk of postoperative hypocalcemia by comparing patients who received zoledronate with those who did not. In addition, because the interval between zoledronate administration and surgery varied substantially in clinical practice, the infusion-to-surgery interval was evaluated as an exploratory, hypothesis-generating endpoint among BP-treated patients.

## 2. Materials and Methods

### 2.1. Study Design and Setting

This retrospective comparative cohort study included patients undergoing parathyroidectomy for PHPT between 2024 and 2025. Seventy-three patients with biochemically and radiologically confirmed PHPT were included. Patients with secondary or tertiary hyperparathyroidism, prior parathyroid surgery, incomplete follow-up data, or persistent postoperative hypercalcemia suggestive of incomplete resection were excluded. Patients with negative or discordant preoperative localization studies and those who required four-gland exploration during surgery were also excluded. All patients underwent surgical excision of a single parathyroid adenoma without concomitant thyroid surgery, and adenoma localization was determined preoperatively using standard imaging methods.

Patients who received intravenous zoledronate prior to surgery were categorized as the BP-positive group whereas those without BP exposure were categorized as the BP-negative group. The study was conducted in accordance with the Declaration of Helsinki and was approved by the local institutional ethics committee (Approval number/date: 0145/13 March 2025).

### 2.2. Data Collection

Demographic and clinical variables, baseline biochemical parameters, adenoma size, and DXA T-scores were extracted from medical records. Biochemical variables included serum calcium, phosphorus, magnesium, ALP, PTH, vitamin D, creatinine, blood urea nitrogen (BUN), and urinary calcium excretion. Markers of bone turnover beyond ALP, such as C-terminal telopeptide or procollagen type, and nuclear bone scintigraphy were not routinely measured in this retrospective cohort, and therefore, could not be included in the analysis.

Patients who received preoperative zoledronate were identified retrospectively. Zoledronate was administered for significant severe hypercalcemia to optimize patients for surgery rather than according to predefined study protocol or specifically as prophylaxis against hungry bone syndrome. The pretreatment calcium range prompting zoledronate use was 12.0–15.6 mg/dL. Because the interval between bisphosphonate infusion and surgery varied among patients’ biochemical parameters obtained after zoledronate administration but before surgery were considered the preoperative baseline for perioperative analyses in BP-treated patients. Thus, calcium values prior to BP infusion characterized the indication for treatment, whereas post-infusion preoperative values reflected the physiological state closest to surgery and were used for comparisons with postoperative status. Among BP-treated patients, the interval between zoledronate infusion and surgery was recorded in days.

Postoperative calcium measurements were retrieved from the perioperative period and during follow-up after discharge.

### 2.3. Outcomes and Definitions

The primary outcome was postoperative hypocalcemia, defined as any albumin-corrected serum calcium < 8.5 mg/dL documented during the postoperative period. Patients meeting this criterion were clinically reviewed for hypocalcemia phenotypes (transient hypocalcemia, HBS, early severe hypocalcemia, and hypoparathyroidism) based on biochemical patterns and PTH levels; these phenotypes were used for descriptive characterization rather than as primary endpoints. A temporary decrease in serum calcium below 8.5 mg/dL after surgery that resolves spontaneously or with short term calcium supplementation, without evidence of permanent hypoparathyroidism is considered as transient postoperative hypocalcemia. Hypocalcemia occurring in association with any phenotype was considered as the primary outcome of this study. Surgical cure was defined as postoperative normalization and maintenance of serum calcium levels within the normal range throughout the 6-month follow-up period.

### 2.4. Statistical Analysis

Normality was assessed using visual inspection and the Shapiro–Wilk test. Because distributions were non-normal, continuous variables are presented as median (minimum–maximum) and compared using the Mann–Whitney U test; categorical variables were compared using Fisher’s exact test. The primary outcome was postoperative hypocalcemia (yes/no). Unadjusted associations were examined using contingency tables.

To evaluate independent associations, logistic regression models were constructed and restricted a priori to two predictors due to the limited number of hypocalcemia events, to minimize overfitting. ALP was selected for the primary adjusted model based on biological plausibility and prior evidence [6,11]. A separate exploratory sensitivity model was built including urinary calcium. Model calibration was assessed with the Hosmer–Lemeshow test. A two-sided *p* value < 0.05 was considered statistically significant.

## 3. Results

Postoperative hypocalcemia occurred in 8 of 73 patients (11%), and all patients achieved surgical cure during post-operative follow-up period. Baseline characteristics by BP exposure (BP-positive vs. BP-negative) are shown in Table 1. BP-treated patients exhibited selectively higher PTH levels, lower phosphorus levels, and lower lumbar spine BMD, whereas preoperative calcium, ALP, adenoma size, vitamin D level, femoral T-score, and renal function parameters did not differ significantly between groups.

Baseline characteristics stratified by hypocalcemia status are summarized in Table 2. Patients who developed hypocalcemia more frequently received preoperative zoledronate than those without hypocalcemia (75.0% vs. 24.6%, *p* = 0.008) (Appendix A Table A1). Severe hypocalcemia; one case hungry bone syndrome and one case early severe hypocalcemia occurred in the bisphosphonate-treated group. The hypocalcemia group had higher ALP levels (152 IU/L vs. 92 IU/L, *p* = 0.006) and higher urinary calcium excretion (470 mg/day vs. 336 mg/day, *p* = 0.038), while preoperative calcium did not differ between groups (*p* = 0.922). As expected, in the hypocalcemia group the lowest postoperative calcium levels were ranging between 6.20–8.40 mg/dL and were significantly lower (*p* < 0.001).

In the primary multivariable logistic regression model adjusting for ALP (Table 3), ALP was independently associated with hypocalcemia (OR 1.016 per unit increase, 95% CI 1.001–1.031; *p* = 0.040), the association between BP exposure and hypocalcemia was not statistically significant after adjustment (OR 4.81, 95% CI 0.74–31.30; *p* = 0.100).

Due to a limited number of outcome events relative to the number of candidate predictors, urinary calcium could not be included in the primary multivariable model and was therefore evaluated in an exploratory analysis, presented in Appendix A Table A2. This analysis was not part of the original model specification and should be considered hypothesis-generating.

In an exploratory analysis restricted to BP-treated patients, the interval between zoledronate administration and surgery was shorter in those who developed hypocalcemia (median 10 days vs. 72 days; *p* = 0.0045; Table 4).

## 4. Discussion

In this retrospective cohort of patients undergoing parathyroidectomy for PHPT, postoperative hypocalcemia occurred in 11% of cases and was associated with markers of increased bone turnover, particularly higher ALP levels. Hypocalcemia was more frequent among patients treated preoperatively with zoledronate; however, this association was attenuated after adjustment for ALP. This finding should be interpreted in the context of confounding by indication and the non-randomized use of zoledronate, which was administered to patients with severe hypercalcemia requiring optimization before surgery.

Post-parathyroidectomy hypocalcemia encompasses heterogeneous entities, including HBS, early severe hypocalcemia, and hypoparathyroidism [6,7,8,9]. In PHPT, HBS is generally less frequent than in secondary hyperparathyroidism, and reported incidence varies depending on diagnostic criteria [10]. In our cohort, one patient met criteria for HBS and no cases of hypoparathyroidism were observed. Only one patient experienced early severe hypocalcemia, indicating that most hypocalcemia events were transient and clinically manageable. The low frequency of severe phenotypes in our study may be related to consistent adenoma-only surgery without concurrent thyroidectomy and the involvement of high-volume surgeons.

The literature on preoperative BP use in PHPT is conflicting and remains inconclusive. Several observational studies and meta-analyses have suggested that BPs may reduce HBS incidence and calcium replacement requirements [14,15,18], whereas other reports have described postoperative hypocalcemia with zoledronate exposure [16,17]. In our study, BP-treated patients did not differ from untreated patients in post-infusion preoperative calcium, ALP, adenoma size, vitamin D level, femoral BMD, or renal function parameters; however, they had selectively higher PTH levels, lower phosphorus levels, and lumbar BMD (Table 1). These findings indicate selected biochemical and skeletal differences rather than a uniformly different risk profile. Higher PTH and skeletal involvement overlap with previously reported risk profiles for postoperative hypocalcemia and HBS, whereas the predictive roles of vitamin D status, phosphorus, and site-specific BMD are less consistent across studies [6,7,19]. Although vitamin D levels did not differ significantly between BP-treated and untreated patients, median values in both groups were within the insufficient range; therefore, future prospective studies should standardize vitamin D assessment and replacement when evaluating zoledronate timing and postoperative hypocalcemia.

Within this context, the identification of alkaline phosphatase as a possible predictor underscores the role of bone turnover in postoperative calcium dynamics. BP exposure was not statistically significantly associated with hypocalcemia; however, the study was underpowered to detect modest independent effects.

The acute postoperative shift from osteoclastic to osteoblastic bone activity, leading to rapid skeletal uptake of calcium, phosphorus, and magnesium, supports the relevance of bone turnover markers and preoperative skeletal status in predicting postoperative hypocalcemia. Prior single-center studies have defined HBS cases by presence of hypocalcemia with hypophosphatemia and normal or elevated PTH levels occurring postoperative day 4 and onward, and have linked it to high–bone-turnover and severe biochemical disease, characterized by higher calcium, PTH, and ALP levels [6,11,20]. Our findings appear consistent with this framework, as ALP was associated with postoperative hypocalcemia in our cohort, whereas zoledronate was not statistically significant after adjustment. Given that only eight cases of hypocalcemia were observed, the study is underpowered to detect less frequent associations. The resulting wide confidence intervals indicate that, although no statistically significant association was identified, a clinically meaningful relationship between zoledronate and hypocalcemia cannot be excluded.

Importantly, our analysis also provides a practical observation regarding treatment timing: among BP-treated patients, hypocalcemia occurred more frequently when zoledronate was administered closer to surgery (median 10 vs. 72 days). This analysis was not a primary objective of the study and should be considered exploratory. Nevertheless, the finding suggests that the infusion-to-surgery interval may influence postoperative calcium dynamics, representing a potentially modifiable perioperative factor and merits further investigation. A major strength of our study is the relatively large number of patients receiving preoperative zoledronate compared with existing literature, which has largely been limited to small case series. This larger sample size enhances the robustness and clinical relevance of our finding while still requiring cautious interpretation because of small number of hypocalcemia events.

This study provides comparative data on preoperative zoledronate exposure in PHPT using a clearly defined surgical cohort with uniform adenoma-only surgery and detailed biochemical and skeletal profiling. Important limitations include the retrospective design, the small number of hypocalcemia events, and the non-standardized clinical criteria for zoledronate use. Because zoledronate was administered for clinically significant severe hypercalcemia according to physician judgement rather than predefined protocol, residual confounding by indication cannot be excluded. Although we used a parsimonious regression model to minimize overfitting, the limited number of hypocalcemia events restricted the number of variables that could be included, and important factors such as PTH and bone mineral density could not be fully adjusted for. In addition, bone turnover markers beyond ALP, such as C-terminal telopeptide or procollagen type 1, N-terminal telopeptide and nuclear bone scintigraphy were not routinely available. Therefore, exploratory findings, particularly the infusion-to-surgery timing analysis, should be interpreted cautiously and validated in larger prospective cohorts.

From a clinical standpoint, BPs remain valuable for managing severe hypercalcemia and osteoporosis in PHPT [2,4,5]. Our findings suggest that perioperative risk stratification using bone turnover markers may be useful in reducing hypocalcemia events. However, these observations should not be interpreted as evidence to withhold indicated BP therapy; rather, they support individualized postoperative monitoring and prophylactic strategies in higher-risk patients [21,22]. Because zoledronate was used to optimize severe hypercalcemia, the present study cannot validate or reject specific treatment-selection criteria for bisphosphonate use before parathyroidectomy. Instead, our findings suggest that future protocols should evaluate both treatment indication and treatment timing, including whether a longer interval between zoledronate infusion and surgery reduces hypocalcemia risk while preserving perioperative safety. In selected cases, preoperative medical control of hypercalcemia with zoledronate therapy may serve as a supportive strategy to improve perioperative safety, facilitate anesthetic management, and allow adequate time for preoperative localization and optimization. Collectively, our results contribute to the growing body of literature informing pragmatic, risk-adapted perioperative strategies rather than uniform treatment approaches in patients with severe hypercalcemia.

## 5. Conclusions

In patients undergoing parathyroidectomy for PHPT, postoperative hypocalcemia occurred in 11% of cases and was associated with higher ALP levels, supporting the role of increased bone turnover. Although hypocalcemia was more frequent among patients receiving preoperative zoledronate, this association was attenuated after adjustment and may reflect underlying treatment selection and disease characteristics. Given the limited number of events, an independent effect of zoledronate cannot be excluded. Among BP-treated patients, a shorter interval between zoledronate administration and surgery was associated with hypocalcemia in an exploratory analysis, suggesting that treatment timing may influence postoperative calcium dynamics and warrants further prospective evaluation. These findings suggest that perioperative risk stratification using bone turnover markers and consideration of treatment timing may help guide postoperative monitoring and management, while further prospective studies are needed to clarify the role of preoperative bisphosphonate therapy.

## Figures and Tables

**Table 1:** Preoperative baseline characteristics according to bisphosphonate (BP) use.

Variable	BP-Negative (n = 51)	BP-Positive (n = 22)	*p* Value
Age, years	56.0 (23.0–87.0)	60.0 (21.0–77.0)	0.301
Preoperative calcium, mg/dL	11.50 (9.80–13.30)	11.45 (8.80–12.00)	0.292
Lowest postoperative calcium, mg/dL	9.20 (8.10–10.40)	8.95 (6.20–10.20)	0.278
Preoperative phosphorus, mg/dL	2.40 (1.50–3.50)	2.15 (1.20–2.80)	**0.007**
Preoperative magnesium, mg/dL	2.00 (1.50–2.50)	2.05 (1.23–2.40)	0.618
PTH, ng/L	159 (62–608)	285 (104–970)	**0.002**
BUN, mg/dL	14.0 (7.0–32.0)	13.5 (7.0–35.0)	0.450
Creatinine, mg/dL	0.80 (0.50–1.60)	0.88 (0.58–1.40)	0.488
ALP, IU/L	97 (49–298)	112 (44–212)	0.458
Urinary calcium, mg/day	333.5 (115–767)	350.5 (120–812)	0.092
Vitamin D, ng/mL	16.7 (5.26–55.4)	17.5 (5.0–35.8)	0.756
Adenoma size, mm	13.0 (3.0–31.0)	16.0 (6.0–35.0)	0.386
DXA femur T-score	−1.30 (−3.20–0.80)	−1.45 (−5.20–0.00)	0.212
DXA lumbar T-score	−2.10 (−4.40–2.00)	−2.80 (−5.50–−1.50)	**0.005**

Footnote: Data are presented as median (minimum–maximum). BP use reflects preoperative bisphosphonate exposure. Preoperative values used for group comparisons were obtained closest to surgery; in BP-positive patients, these values were measured after zoledronate infusion but before surgery. The pretreatment calcium range (12.0–15.6 mg/dL) was used only to characterize the indication for zoledronate therapy. Mann–Whitney U test. Bold *p* values indicate statistically significant between-group differences (*p* < 0.05).

**Table 2:** Preoperative baseline characteristics according to hypocalcemia occurrence.

Variable	Hypocalcemia (n = 8)	No Hypocalcemia (n = 65)	*p* Value
Age, years	56.0 (50.0–74.0)	56.0 (21.0–87.0)	0.979
Bisphosphonate use, n (%)	6 (75.0)	16 (24.6)	0.008 *
Preoperative calcium, mg/dL	11.40 (10.7–12.00)	11.50 (8.8–13.30)	0.922
Lowest postoperative calcium, mg/dL	8.30 (6.20–8.40)	9.30 (8.50–10.40)	<0.001
Preoperative phosphorus, mg/dL	2.40 (1.20–3.50)	2.05 (1.20–3.50)	0.271
Preoperative magnesium, mg/dL	2.12 (1.23–2.20)	2.00 (1.50–2.50)	0.133
PTH, ng/L	267 (89–970)	166 (62–757)	0.108
BUN, mg/dL	13.5 (7.0–20.0)	14.0 (7.0–35.0)	0.394
Creatinine, mg/dL	0.75 (0.58–1.20)	0.84 (0.50–1.60)	0.187
ALP, IU/L	152 (113–200)	92 (44–298)	**0.006**
Urinary calcium, mg/day	470 (260–812)	336 (115–767)	**0.038**
Vitamin D, ng/mL	16.7 (5.26–55.40)	18.5 (5.00–25.00)	0.565
Adenoma size, mm	17.5 (10.0–28.0)	13.0 (3.0–35.0)	0.239
DXA femur T-score	−1.40 (−5.20–0.80)	−1.50 (−2.40–−0.20)	0.714
DXA lumbar T-score	−2.65 (−3.90–−1.50)	−2.30 (−5.50–2.00)	0.725

Footnote: Mann–Whitney U test. Data are presented as median (minimum–maximum) due to non-normal distributions. Hypocalcemia was defined as postoperative serum calcium level below the institutional reference range. In BP-positive patients preoperative biochemical values refer to post infusion, presurgical measurements. Bold *p* values indicate statistically significant between-group differences (*p* < 0.05). * Fisher’s exact test (see Appendix A Table A1).

**Table 3:** Primary multivariable logistic regression analysis for hypocalcemia.

Variable	OR	95% CI	*p* Value
BP use (yes vs. no)	4.81	0.74–31.30	0.100
ALP (per unit increase)	1.016	1.001–1.031	0.040

Footnote: The model was restricted to two predictors due to the limited number of hypocalcemia events to minimize overfitting. Model statistics: Nagelkerke R^2^ = 0.257; Hosmer–Lemeshow *p* = 0.357.

**Table 4:** Time interval between bisphosphonate initiation and surgery among bisphosphonate-treated patients.

Outcome Group	n	Median (Days)	IQR (Days)	Mean ± SD (Days)	Range (Days)	*p*
Hypocalcemia (+)	6	10	4–18	16.0 ± 18.4	2–51	0.0045
Hypocalcemia (−)	16	72	36–110	82.3 ± 58.5	5–210	

Footnotes: Analysis was restricted to patients receiving bisphosphonate therapy and considered exploratory because the infusion to surgery time was not a primary endpoint. Data are presented as median (interquartile range), unless otherwise indicated. Comparisons were performed using the Mann–Whitney U test due to non-normal distributions.

## Data Availability

The datasets generated during and/or analyzed during the current study are available from the corresponding author on reasonable request.

## Abbreviations

The following abbreviations are used in this manuscript:

PHPT	Primary hyperparathyroidism
BP	Bisphosphonate
PTH	Parathyroid hormone
BMD	Bone mineral density
HBS	Hungry bone syndrome
ALP	Alkaline phosphatase
DXA	Dual-energy X-ray absorptiometry
BUN	Blood urea nitrogen

## Appendix A

**Table A1:** Unadjusted association between bisphosphonate use and hypocalcemia.

	Hypocalcemia	No Hypocalcemia	*p*
BP use (yes)	6 (27.3%)	16 (72.7%)	0.008
BP use (no)	2 (3.9%)	49 (96.1%)	

Fisher’s exact test. Given the limited number of hypocalcemia events (n = 8), these comparisons should be interpreted with caution.

**Table A2:** Exploratory logistic regression adjusted for urinary calcium.

Variable	OR	95% CI	*p* Value
BP use (yes vs. no)	4.14	0.65–26.50	0.133
Urinary calcium (per unit increase)	1.004	0.999–1.009	0.092

Footnote: This exploratory model was restricted to two predictors because of the limited number of hypocalcemia events. Model statistics: −2 log likelihood = 35.40; Nagelkerke R^2^ = 0.238; Hosmer–Lemeshow *p* = 0.506. Results should be interpreted cautiously and are intended as a sensitivity analysis.
